# Antimicrobial resistance as a multicultural challenge: a decolonized approach to science communication in the Global South

**DOI:** 10.1093/jacamr/dlag038

**Published:** 2026-03-19

**Authors:** Han Thai Dong Tran, Marina Joubert, Mary Chambers

**Affiliations:** Oxford University Clinical Research Unit, 764 Vo Van Kiet, Cho Quan Ward, Ho Chi Minh City, Vietnam; Nuffield Department of Medicine, University of Oxford, Henry Wellcome Building for Molecular Physiology, Old Road, Oxford OX3 7BN, UK; Centre for Research on Evaluation, Science and Technology (CREST), Stellenbosch University, Krotoa Building, Stelenbosch Campus, Stellenbosch Central, Stellenbosch, South Africa; Oxford University Clinical Research Unit, 764 Vo Van Kiet, Cho Quan Ward, Ho Chi Minh City, Vietnam; Nuffield Department of Medicine, University of Oxford, Henry Wellcome Building for Molecular Physiology, Old Road, Oxford OX3 7BN, UK

## Abstract

Antimicrobial resistance (AMR), or drug-resistant infection, is a serious threat to global well-being, impacting human, animal and environmental health, and is predicted to cause more deaths than cancer by 2050 (10 million deaths annually). AMR is often termed a super-wicked problem that prevents straightforward solutions due to the systemic, multi-sectoral and multifactorial issues underlying it, posing a dilemma for AMR communication in LMICs. Antimicrobial stewardship, which consists of coordinated efforts to optimize antimicrobial use to reduce AMR, and communication for antimicrobial stewardship behaviours, are hindered by many cultural factors: subjective norms, medical pluralism, hierarchical culture, etc. This Viewpoint suggests ways and examples for how a decolonized approach can support communication around a multicultural issue such as AMR, for understanding drivers and exploring locally relevant solutions. These include recognizing systemic barriers, social norms and collective values, challenging the universalism of Western science to value local knowledge and practices, promoting local ownership of intervention design and knowledge generation, adapting to local structural norms and hierarchies, and being aware of the language problem of AMR. It is imperative that locally contextualized capacity strengthening is available to empower Global South science communication actors (researchers, academics and practitioners).

Antimicrobial resistance (AMR) is a major global health challenge as once-effective treatments fail against common infections, with the heaviest burden in low- and middle-income countries (LMICs).^1,2^ (This Viewpoint uses examples and suggestions related to antibiotic use to discuss the issue around AMR communication. However, the same challenges may also apply to other types of antimicrobials—i.e. antifungals, antiparasitics and antivirals.) Although public communication is vital to address AMR, current efforts often emphasize awareness-raising but lack behavioural and theoretical underpinnings and actionable messages, particularly for Global South audiences.^3–6^ This Viewpoint proposes a decolonized approach to AMR communication. In this context we refer to decolonization as recognizing historical and political influences that have marginalized certain groups and ideas from knowledge creation endeavours; thus, decolonized science communication is receptive towards local context and insights. Although applicable globally, we are considering this primarily from a Southeast/East Asian perspective.

## Recognizing structural and systemic barriers

Communication interventions aiming at raising awareness of AMR and individual responsibility are likely to be insufficient.^7^ The reasons for taking or prescribing antibiotics stem from larger systemic issues, limiting the capacity of individuals to act, and creating dilemmas for AMR communication. Community members in low-resourced settings may not be able easily to act upon what is communicated to them: ‘Do not save leftover antibiotics’. People see self-medication as the solution for overcoming barriers related to distance, cost and limited resources for accessing formal healthcare.^8,9^ The accessibility of antibiotics in LMICs is regarded by many as a quick fix for the weak infrastructures of healthcare and infection prevention.^10–12^ Healthcare professionals in LMICs encounter socioeconomic barriers that limit their role for antimicrobial stewardship, and consequently AMR communication. These barriers include diagnostic uncertainty, empathy for patients’ financial situations, and conflicts between personal interest and duties.^13–16^ High patient–doctor ratios and lack of privacy in clinical settings prevent thorough examinations and conversations about treatment options.^8,17^ Decolonized communication strategies would be mindful of this dilemma, moving from mere dissemination of unactionable messages. Considering a lack of scientific consensus about what these ‘actionable messages’ should be in different settings, decolonized communication would foster in-depth engagement for context-sensitive messages and solutions.

## Addressing social norms and collective values

In collectivist cultures, such as those prevalent in Asia, individual behaviour is strongly influenced by social norms and expectations.^18^ Perceived social pressures about using antibiotics as a sign of ‘proper’ childcare are directly linked to parental self-medication of antibiotics.^9,18^ Individuals may find it difficult to act upon what is communicated to them about AMR, especially if those messages deviate from or challenge accepted social norms. Locally relevant AMR communication would recognize the importance of group harmony, conformity, and the influence of community leaders in some cultures.^19^ Messaging that aligns with these collective values—rather than focusing solely on individual responsibility—will be more effective in changing behaviours related to antibiotic use.

## Valuing local knowledge and practices

Current global health discourse around AMR is primarily based on Western modern biomedicine knowledge and solutions relevant to high-income countries.^20^ In Southeast Asia, traditional and alternative medicines coexist with biomedicine (i.e. medical pluralism), combining principles and practices of these disciplines, thus influencing people’s use of antibiotics.^9,20^ A decolonized approach would challenge the hierarchy of knowledge to expand understanding of AMR by revealing such local perspectives. Traditional knowledge and lived experience should be recognized as alternative forms of knowledge,^20^ and could feed into locally relevant solutions. Communication that values local knowledge and practices fosters trust for local communities.

## Promoting co-design and emphasizing knowledge co-production for local ownership

Community engagement is posed as the key to facilitate AMR behaviour change because it involves community members in co-designing solutions.^21^ A decolonized approach encourages dialogues, not only to gain trust and acceptance, but also to challenge the hierarchy of knowledge. More than trying to get a message across, it is receptive to local insights to help communicators, public health practitioners and researchers learn about the sociocultural context of AMR, better define the research problem, and interpret findings grounded in local realities.^20,22^ In other words, the knowledge that feeds into the AMR discourse is co-produced and locally owned.

## Adapting to local norms and hierarchies

Western models of science communication often assume shared decision-making and flat hierarchies, but some cultures, such as those in Southeast Asia, are hierarchical. Cai *et al*.^23^ have reported challenges implementing a bottom-up approach for community engagement in the context of Vietnam, as community stakeholders were used to passively following the top-down instructions. Additionally, in such hierarchical cultures the feasibility of shared-decision making in consultation and patient advisory roles for antimicrobial stewardship, as proposed for high-income contexts,^24–27^ is questionable. A didactic communication style between doctors and patients is the norm, but places patients in a passive position, waiting for cues to engage in conversations or avoiding asking questions for fear of being misunderstood or seeming to mistrust healthcare workers.^9,28^ Rather than imposing Western ideals of patient autonomy that may not resonate or be feasible, decolonized communication means designing interventions that respect these social structures and take additional steps to build consensus on using novel approaches.

## Being mindful of the lack of local language for AMR terminologies

In many countries there is no direct translation for antibiotics and AMR terminologies; or the local translation does not convey the concepts accurately, leading to misconception and misuse.^9,15,29–31^ Community members may use colloquial terms and are confused by official terms.^15,31^ A decolonized approach would be mindful of this, using accessible language and additional explanation to ensure understanding of the issue.

## A way forward

We propose a decolonized approach that is careful to avoid blaming individuals for behaviours shaped by broader social, economic and systemic factors. Instead, our approach seeks to motivate individual and collective responses by empowering communities with information that is relevant and actionable within their specific contexts. By questioning the assumption that Western scientific knowledge and communication models are universally applicable, decolonization opens the door to more pluralistic, context-sensitive and equitable approaches. It also promotes mutual learning between global and local actors.

There is little guidance on AMR communication for Global South researchers. Decolonization means empowering them with the capacity, opportunity and motivation to lead this work. Training grounded in behaviour-change theory is needed to support local actors in realizing their identity and agency for AMR communication. If researchers are actively involved in key decisions for communicating AMR with local audiences, using approaches that incorporate sociocultural factors and local knowledge, responses to AMR could be more meaningful, ethical and actionable.
